# A Framework to Secure the Development and Auditing of SSL Pinning in Mobile Applications: The Case of Android Devices

**DOI:** 10.3390/e21121136

**Published:** 2019-11-21

**Authors:** Francisco José Ramírez-López, Ángel Jesús Varela-Vaca, Jorge Ropero, Joaquín Luque, Alejandro Carrasco

**Affiliations:** 1 Departamento de Tecnología Electrónica, Universidad de Sevilla, 41012 Sevilla, Spain; framirez4@us.es (F.J.R.-L.); jropero@us.es (J.R.); jluque@us.es (J.L.); 2 Departamento de Lenguajes y Sistemas Informáticos, Universidad de Sevilla, 41012 Sevilla, Spain; ajvarela@us.es

**Keywords:** SSL pinning, security, mobile applications, Android, auditing, vulnerabilities, OWASP

## Abstract

The use of mobile devices has undergone rapid growth in recent years. However, on some occasions, security has been neglected when developing applications. SSL/TLS has been used for years to secure communications although it is not a vulnerability-free protocol. One of the most common vulnerabilities is SSL pinning bypassing. This paper first describes some security controls to help protect against SSL pinning bypassing. Subsequently, some existing methods for bypassing are presented and two new methods are defined. We performed some experiments to check the use of security controls in widely used applications, and applied SSL pinning bypassing methods. Finally, we created an applicability framework, relating the implemented security controls and the methods that are applicable. This framework provides a guideline for pentesters and app developers.

## 1. Introduction

Nowadays, the use of mobile devices is constantly increasing to do the same operations that used to be done using web services less than a decade ago [1,2]. However, it is necessary to provide the same security solutions in both environments since both operations are equally critical. SSL/TLS (Secure Socket Layer/Transport Layer Security) technology has been widely used as the basis to secure many aspects of the Internet, such as securing HTTP protocol [3].

In 2018, 1.54 billion smartphones were sold [4]. Thus, the rapid growth in the number of smartphones has led to an increase in security threats related to them [5]. Day by day, we read cases of users who have been scammed through the use of mobile applications [6]. For example, users may download some modified version of an application (hereinafter, app) that is not controlled by the owner. In some cases, access to sensitive information by other users of the app has also been detected. This is because many of the controls that have been applied in the web environment have not been considered in the mobile environment. Moreover, several mobile apps do not even implement SSL/TLS validations [7]. Even when using SSL/TLS, apps may have vulnerabilities, especially to Man-in-the-middle (MiTM) attacks [8]. Other attacks, such as replay attack, eavesdropping, and session hijacking, are also common [3]. Security controls such as SSL pinning are desirable [9] but always optional. SSL pinning, also known as certificate pinning or SSL/TLS validations, allows clients to have greater confidence that the certificate used by a server is not compromised [10]. Nevertheless, it is also possible to circumvent SSL/TLS validations [7]. Thus, SSL pinning can be skipped.

Trying to consider the particularities of mobile apps, the OWASP (Open Web Application Security Project) Mobile Application Security Verification Standard (MASVS) is an attempt to standardise these requirements using verification levels that fit different threat scenarios [11]. One of the most important challenges in mobile app security is to protect data flows over insecure communication channels [12]. Insecure communications include poor handshaking, incorrect SSL versions, weak negotiation or clear text communication of Personally Identifiable Information (PII) [13]. In this paper, we explain how an app can be fortified considering some security controls, shielding some aspects to avoid attacks.

This paper first analyses the vulnerabilities of SSL/TLS implementations and the necessity of SSL pinning techniques. Then, we propose a set of security controls that can prevent an app from suffering SSL pinning bypassing. Afterwards, we selected a set of popular Android apps to check if the latter bypassing methods, described in [7] are still valid. We also propose two new bypassing methods, and we have applied them to the app set. Finally, we have defined a framework for the applicability of bypassing methods depending on the security controls that have been applied by the app developers.

The rest of the paper is structured as follows. Section 2 deals with the background of this paper. Concepts such as the OWASP Mobile Testing Guide, the SSL/TLS protocol, the SSL pinning technique and the problem of bypassing of SSL pinning are introduced. Section 3 presents a set of security controls to protect apps against bypassing methods. In Section 4, we evaluate our approach by providing experiments. The obtained results are analysed in Section 5. Finally, the paper is summarised, and the conclusions and future work are presented in Section 6.

## 2. Background

This section presents some fundamental concepts to set the context of this paper. First, the OWASP Mobile Testing Guide for mobile applications is introduced, justifying its use as a security model for web applications. Secondly, the operation of SSL/TLS is briefly described. SSL/TLS has been used for years to add an extra layer of security. Finally, some SSL/TLS vulnerabilities are introduced, so that we justify the need of this research.

### 2.1. OWASP Mobile

OWASP is the worldwide organisation responsible for generating a standard for security in web apps [14]. This way, we can find several sources of information and methodologies in the OWASP documentation. The best-known methodology is the so-called Top 10, where the most frequent vulnerabilities are shown. OWASP group develop Top 10 security risks for web, mobile and IoT software [15]. Based on our experience, we choose OWASP Top 10 Mobile as our starting point. Table 1 shows OWASP Mobile Top 10 in December 2016, which is the last update. [13].

As shown, improper platform usage is considered the most relevant security risk. This category covers the security control that is part of the mobile operating system. However, insecure communication ranks #3 in OWASP Top 10, thus it is also quite an important topic to be considered. SSL pinning is included in this category.

Although there are some other methodologies or lists of controls where apps may be reviewed, we focus on the Mobile Application Security Verification Standard (MASVS), which is defined in the OWASP Testing Guide. The OWASP Mobile Testing Guide was recently published in its first version [11]. In this guide, security controls are defined and can be reviewed according to different categories. Every control is described. It is also shown how it can be tested. Finally, the guide sometimes offers a solution to the problem described by the control. However, the solution must be adapted to the system or the client that is audited.

Within the OWASP controls, several control layers must be considered. The utilisation of these layers depends on the app. There are three existing layers, called verification levels: L1, standard security; L2, defense-in-depth; and R, resiliency against reverse engineering and tampering.

All the controls are grouped into categories. Within each category, different security controls must be applied. We focus on category V5, network communication requirements. To secure network communication, we should follow the recommendations shown in Table 2.

To achieve level L1 requirements, Controls 5.1–5.3 must be secured, while Controls 5.4 and 5.5 refer to level L2. However, accomplishing the requirements of Control 5.4 implies accomplishing the three previous controls. Thus, securing Control 5.4 implies achieving L1 requirements. In practice, we can identify this control with SSL pinning.

### 2.2. SSL and SSL Pinning

Secure Socket Layer (SSL) [16] protocol and Transport Layer Security (TLS) [17] protocol are widely used to provide confidentiality, authentication, and integrity in data communications. SSL/TLS provides three main security services: confidentiality, by encrypting data; message integrity, by using a message authentication code (MAC); and authentication, through digital signatures.

SSL/TLS allows the authentication of both parties, server authentication with an unauthenticated client, and total anonymity. The authentication of client and server may be carried out through digital signatures. Nowadays, digital signatures are mostly based on certificates (i.e., X.509 standard) or shared keys. In the case of using certificates, they always have to be verified to ensure proper signing by a trusted Certificate Authority (CA). On the other hand, these protocols also provide anonymous authentication by using Diffie–Hellman for key exchange from SSLv3.0, TLSv1.0 and later versions [18].

SSL/TSL protocol is based on a handshake sequence whose main features [19] are used by client and server, as follows: (1) Negotiate the cipher suite to be used during data transfer, and exchange random numbers (master key); (2) Establish and share a session ID between client and server; (3) Authenticate the server to the client; and (4) Authenticate the client to the server.

Several providers are widely used such as JSSE (Java Security Socket Extension) [20], OpenSSL [21], LibreSSL [22], or GnuTLS [23]. There is even specific hardware with built-in SSL/TLS solutions, such as in iOS devices [24].

As mentioned above, one of the Top-3 risks identified by OWASP is insecure communication due to poor configuration of an SSL/TLS channel. However, SSL/TLS is not a vulnerability-free protocol [25]. SSL/TLS attacks may come from MiTM Attacks, against the handshake or from the SSL implementation. Moreover, it is demonstrated that most of the Android applications use the default SSL implementations provided by Android [25,26]. Similarly, most of the applications are susceptible to be attacked by intercepting communications by MiTM attacks. Thus, SSL/TLS implementations can be broken by MiTM attacks [15]. When the client is in a public network, it is possible for an attacker to be in the middle of the communication and impersonate the server.MiTM attacks occur due to the lack of validation or an incorrect validation in the protocol.

**Figure 1:**
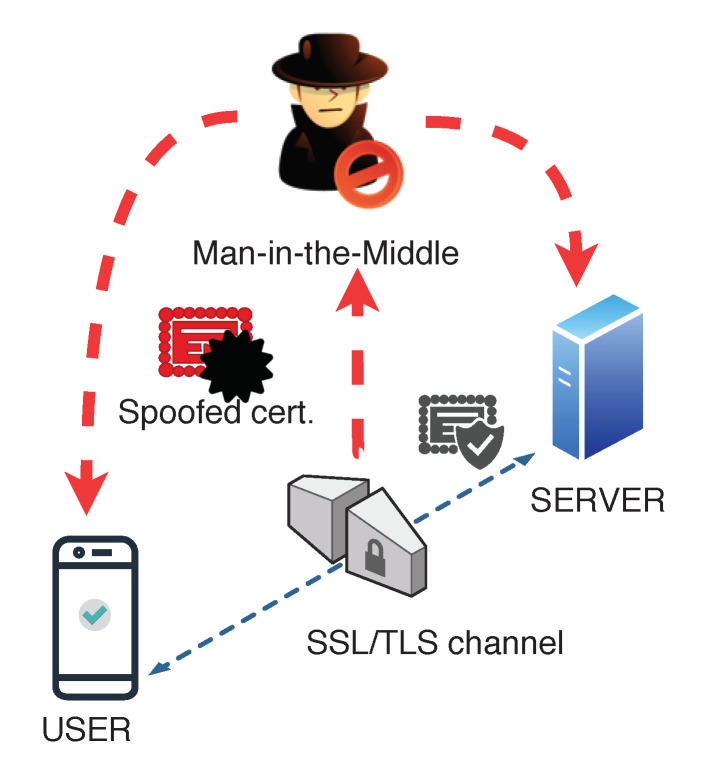
Bypassing SSL/TLS by using spoofed certificates.

In SSL/TLS, certificates are verified to check whether they are signed by proper CA. With a MiTM attack using a spoofed certificate, SSL/TLS may be mislead (see Figure 1). If this is done with a valid certificate, the client’s system checks the certificate and considers it valid. Then, the attacker may capture all the plaintext data that are exchanged between client and server. The certificate is trusted, even though its origin is unknown. Once the certificates are accepted and the handshake is finished, the SSL/TLS communication is established as secure. Meanwhile, a third party is bypassing the channel intercepting and deciphering all the packets in the communication.

The pinning technique or HTTP Public Key Pinning (HPKP) [7] has emerged in the last years as a security control to fortify HTTPS-based pinnings against MiTM attacks. The SSL pinning techniques have been widely used as a complement to enforce the security of SSL/TLS communications for mobile applications [9].

**Figure 2:**
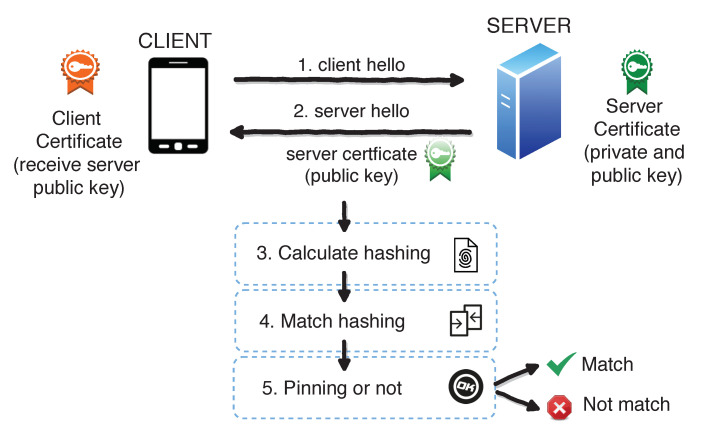
Certificate verification process and pinning.

Figure 2 shows the SSL pinning implementation process, which is divided into two stages. In the first stage, the mobile device must initiate communication with the server. The server responds whether it is active or not (server hello in Figure 2). Then, the client asks for the server’s certification when the server answers with the content of the information of its certificate and public key (verification certificates in Figure 2).

The second stage is called pinning. The mobile device follows a verification process where the certificate is received from the server. When the client receives a message from the server, it checks its authenticity using the server’s public key, which is stored in the client. The received public key has to match the one that is stored. If so, the client opens a negotiation or sends packages signed with that public key. When the client does not coincide, it cuts off the communication. Thus, it does not send anything to the server.

SSL pinning techniques has been proved as good countermeasures against MiTM attacks [27,28,29]. Most of the works in the literature focused on improving SSL/TLS implementations. The utilization of SSL pinning techniques against weak and default implementations of secure communications and MiTM attacks is usual. However, SSL pinning techniques are not invulnerable since it can be circumvented.

### 2.3. Bypassing SSL Pinning

SSL pinning is also vulnerable when it is not well implemented. Although bypassing techniques are quite well-known in the professional world, there are not many studies about bypassing SSL pinning. Andzakovic used reverse engineering to bypass SSL pinning on Android, but the technique was applied to an only app [30]. Sierra and Ramirez [31] also used different techniques to bypass certificate pinning, but only achieved it with dynamic library manipulation. D’Orazio et Choo defined five methods for SSL circumventing on iOS, and their study is the starting point for ours [7]. Finally, Anthi and Theodorakopoulos used some techniques to bypass SSL validations in iOS and Android, but could only bypass SSL in iOS apps [5]. Moreover, several methods can be used to bypassing SSL pinning:

- *Dynamic analysis of code.* The code that is being executed in memory is extracted and its behaviour is low-level analysed, evaluating registers, the data that are loaded in memory and functions, among others. An example of a dynamic analysis tool is SSL Unpinning by Xposed framework. This tool takes advantage of the fact that the code that implements SSL Pinning is known. In general, pinning developers use a well-known or common template. In this case, an attacker may guess this and use SSL Unpinning to bypass SSL Pinning in the pinning stage. In addition, tools such as Frida or Cycript enable the modification of some functions of pinning at runtime.
- *Static analysis of code*. It consists in extracting and analysing the app code when not at runtime. As we explain in Section 3, if pinning does not implement anti-tampering or exceptions for the modification of pinning, SSL Pinning functions can be replaced to bypass the pinning process.

## 3. Security Controls to Protect against Bypassing SSL Pinning

Certain countermeasures, such as security controls, may help an app to avoid the bypassing of the SSL pinning process. With four security measures or controls, apps can be fortified against SSL bypass. Here, we present some good/best practices or guidelines as a set of several steps to implement an adequate security solution to secure SSL Pinning. This way, bypassing SSL pinning is avoided. Although OWASP proposes to use a set of controls to ensure channels of communications, our guideline demonstrates and ensures that, with only three steps, mobile pinnings can be fortified against bypassing SSL pinning, and no more controls need to be checked.

**Figure 3:**
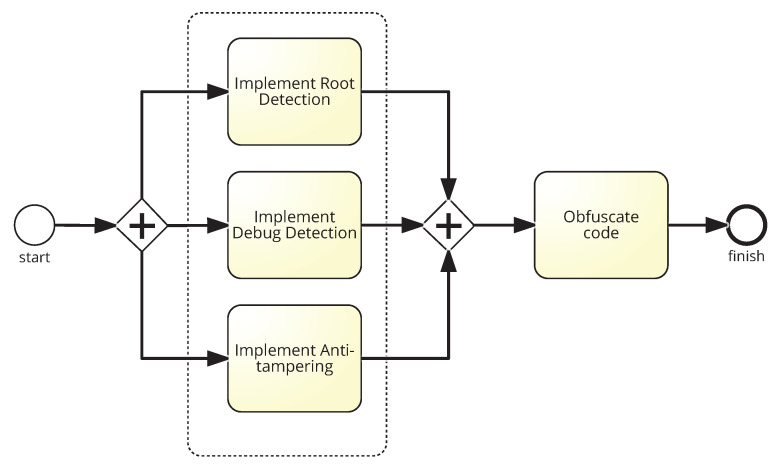
Process to ensure SSL Pinning.

The proposed process (In BPMN, the +−symbol indicates parallel execution of tasks) is shown in Figure 3, which presents the measures that have to be taken to protect an app from SSL pinning bypassing. In the implementation phase of the app, root detection, debug detection and anti-tampering measures must be implemented. Finally, the code must also be obfuscated. All these security measures should be applied to guarantee that SSL pinning is not bypassed. They are described in detail below.

1. **Root Detection** is a security control consisting of checking the blocks of the execution code that turn the pinning into the supervisor mode. Thus, root detection helps prevent the execution of code in supervisor mode. This control improves the effectiveness of reverse engineering and anti-tampering processes.
  There are several ways to detect root [32] but the most common one is checking some files (i.e., binaries and apk files) that are present on a rooted device, such as /system/app/Superuser.apk or /system/bin/su. A piece of the code used to check the root execution on the pinning is given in Code 3.1.

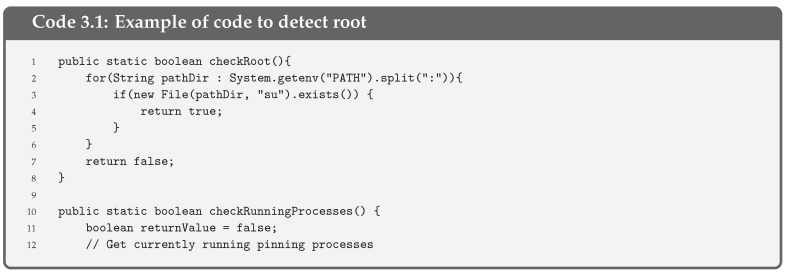

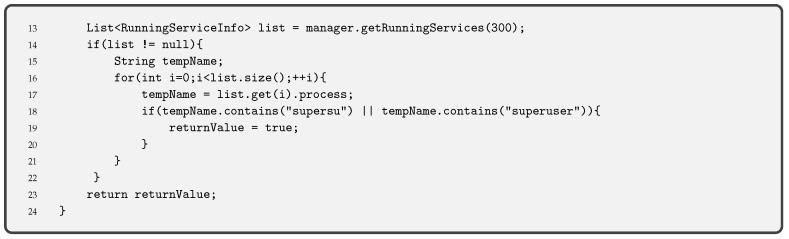

2. **Debug Detection** is used to prevent the app code from being controlled. If a device in debug mode is detected, the execution of the pinning is stopped. This measure stops an attacker from seeing the step-by-step behaviour of our code. Together with the obfuscation of code, described below, it allows hiding the internal functioning of the pinning. Functions such as the one we provide in Code 3.2 are used for Android applications. This piece of code is an adaptation of the guidelines OWASP for debug detection, although there are other ways to detect the debug mode.
  Indeed, it is always possible to compile an app in debug mode (i.e., changing the Android Manifest file and modifying the corresponding label). In this case, the entire device is not in debug mode, but the app. Code 3.2 detects debugging mode with different functions: finding the Debugger flag, detecting debugger, detecting if execution has stopped because of debugging and checking running processes.

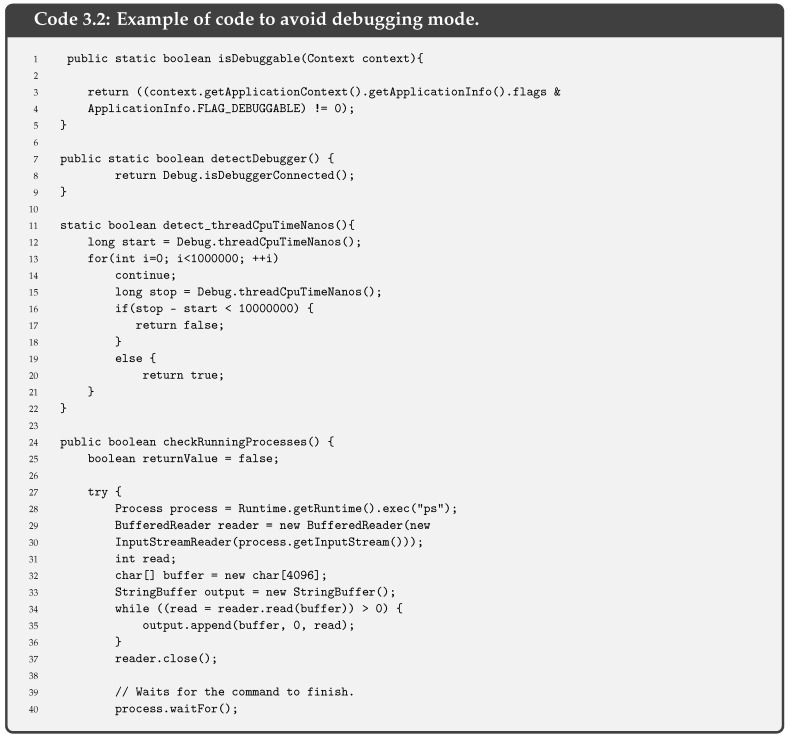

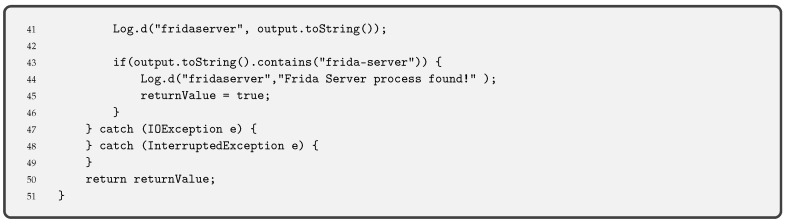

3. **Anti-tampering** solution is an important option since any attacker may decompile an application and recompile it. Modifying some parts of the application [32], as previously explained in the SSL pinning bypass process, an attacker could skip the SSL pinning, and thus make the application invalid.
  Code 3.3 gives an example of the code that can be included in an Android application, particularly in the *onCreate* function within *MainActivity*. In this case, the signature of the application is checked only when the app is started. In iOS, the mechanism is similar, as indicated in the Apple Security Transforms Programming Guide [33].

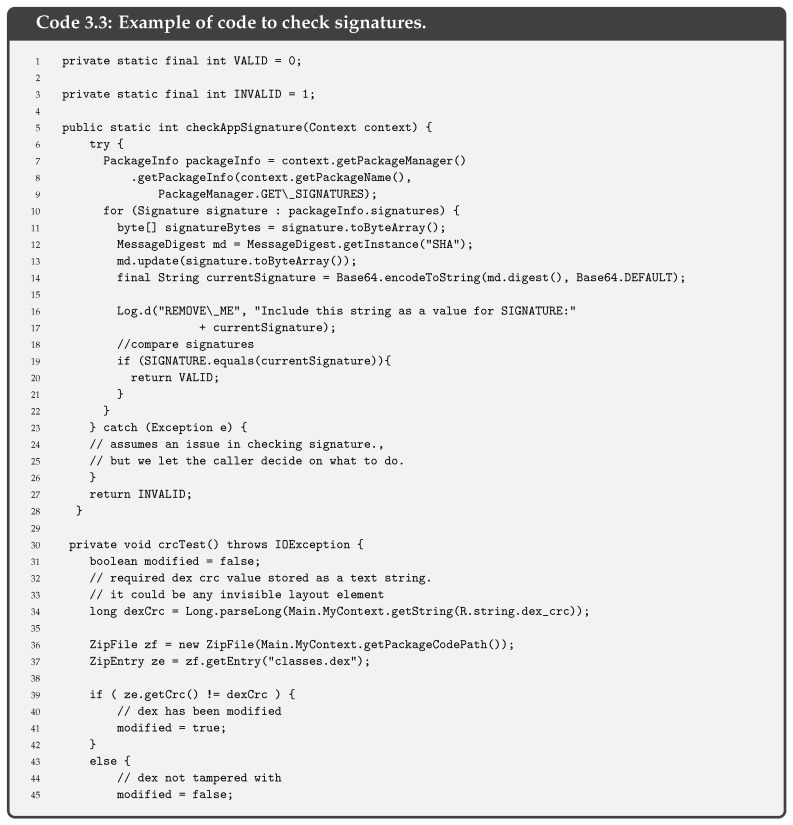

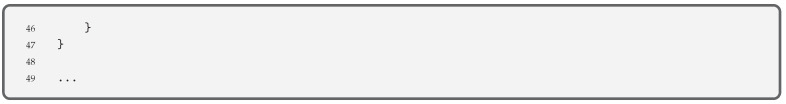

**Figure 4:**
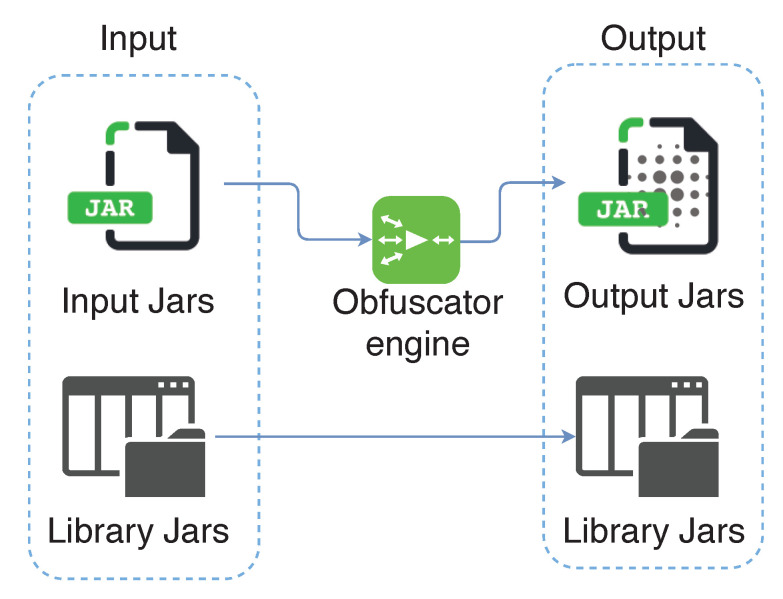
Obfuscation process.

4. **Obfuscate code**. All the measures above do not make sense without preventing any attacker from knowing the code of the app. This way, the analysis of SSL Pinning functions or any of the previous ones that we have presented is more difficult. For this reason, code obfuscation is necessary. There are code obfuscators that convert the existing code into a more illegible one, as seen in Figure 4. Therefore, it is more difficult to detect which are the critical functions of the code for an attacker.
  As may be seen, replacing variables and function names for letters and numbers makes it more difficult to guess what a function does. An example of obfuscation is shown in Code 3.4, where names of variables and functions are hidden.

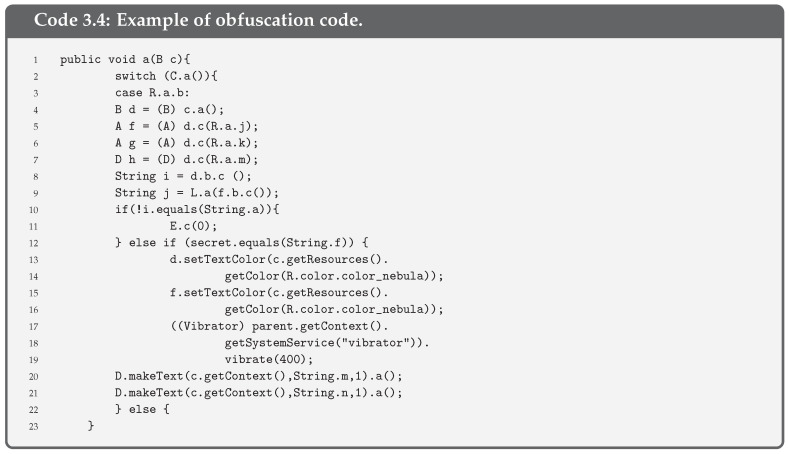

  Numerous tools on Android and iOS allow the obfuscation of code, such as *Proguard* [34] for Android or *iXGuard* [35] for iOS.

As aforementioned, these security controls are an easy way to protect the app against bypassing methods of SSL pinning compared to OWASP guidelines. In spite of security controls, several methods can be used to circumvent the SSL/TLS validations.

## 4. Experiment Setup

Once we have defined some countermeasures to fortify SSL pinning, we must study the methods that can bypass these countermeasures. First, we define a threat scenario. Secondly, we study the current bypassing SSL pinning techniques. Next, we propose new methods of SSL bypassing. Finally, we evaluate our proposal, and provide some guidelines for the correct development and auditing of the applications that use SSL pinning mechanisms.

### 4.1. Threat Scenario

**Figure 5:**
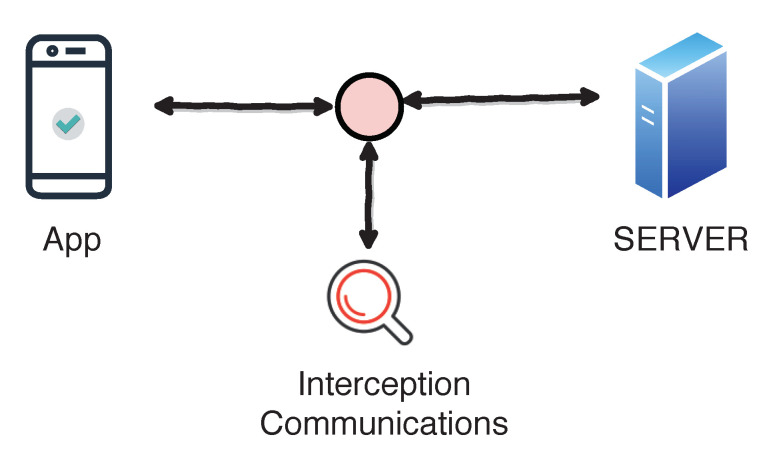
Threat scenario.

It is important to remark that neither the app client nor the server is attacked. Communications are the objective. The attacker could install the app in his/her device, decompile the app, modify the app, compile it again and sign it with his/her own certificate. The server does not verify the new certificate, but the app. The attacker could intercept communications, see how communications are implemented and tamper the app. Thus, the integrity of the information is compromised. Figure 5 represents the threat scenario.

For example, if the client uses the app to make a payment, he/she could bypass SSL pinning, see how communications are implemented (if payment is not ciphered) using a MitM attack and modify the values (paying 10 instead of 100, for instance). Obviously, other features of the app could also be modified.

### 4.2. Current Bypassing Methods

A MiTM attack can break SSL/TLS pinning, as explained in Section 2.3. Tools such as Burp Suite or Charles Proxy may be used for these attacks. D’Orazio and Choo [7] defined five methods to bypass built-in SSL/TLS validations for iOS devices. They attempted to bypass SSL/TLS validations in 40 popular iOS apps but, surprisingly, only 10 of them performed SSL/TLS validations. The five methods that are used are described below:

1. **Method 1:** Change common name (CM) in certificate. It is similar to the MiTM attack described in Section 2.3, with the proxy changing the CA-signed certificate dynamically.
2. **Method 2:** Insert CA in the application. This method modifies the CA signature that is stored inside the application.
3. **Method 3:** Use SSL Unpinning. Root access is necessary in this case. Tools such as Xposed in Android or SSL Kill Switch are used for SSL bypassing. They take advantage of the fact that the code that implements SSL Pinning is known. App developers use a well-known template, so that an attacker may guess it and use these tools to bypass SSL Pinning in the app.
4. **Method 4:** Debugging app. If the app does not implement anti-tampering or exceptions for the modification of the app, SSL Pinning functions can be replaced to bypass the pinning process.
5. **Method 5:** Modify app executable to avoid the SSL pinning process. The modified version of the app is copied to the device to permanently bypass SSL/TLS pinning.

D’Orazio and Choo’s results are shown in Table 3. A checkmark means that the SSL/TLS implementation was bypassed using that method. Otherwise, a dash symbol is used. However, we must highlight two aspects of the results: (1) the authors avoided checking the security controls presented in Section 3; and (2) the tests were only carried out in iOS apps.

### 4.3. New SSL Pinning Bypassing Methods

As a new contribution, we propose two new methods to bypass SSL/TLS validations in Android devices. The first method deals with rooted devices and the second one deals with non-rooted devices. Since the new methods were compared with the ones defined in Section 4.2, we decided to number the new methods as Methods 6 and 7, respectively. Methods 6 and 7 makes circumventing SSL pinning in debug mode possible.

#### 4.3.1. Method 6: Dynamic Debugging of the App in Rooted Devices

**Figure 6:**
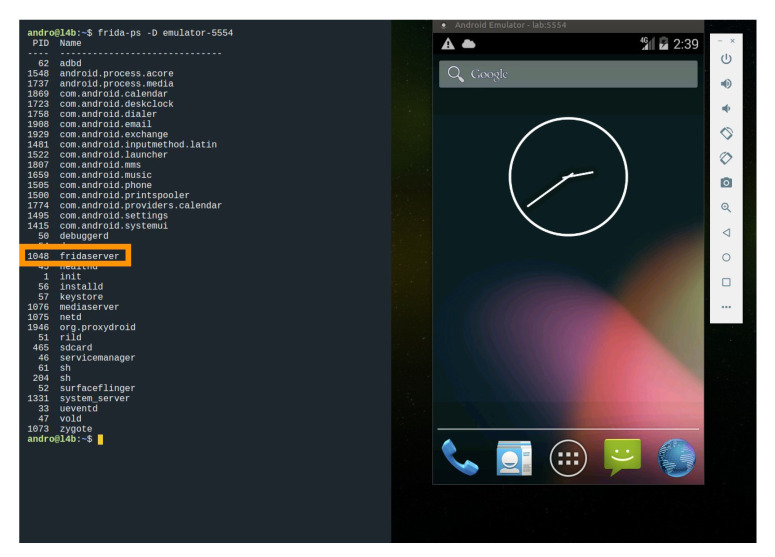
Example of Frida server running in a mobile emulator.

**Figure 7:**
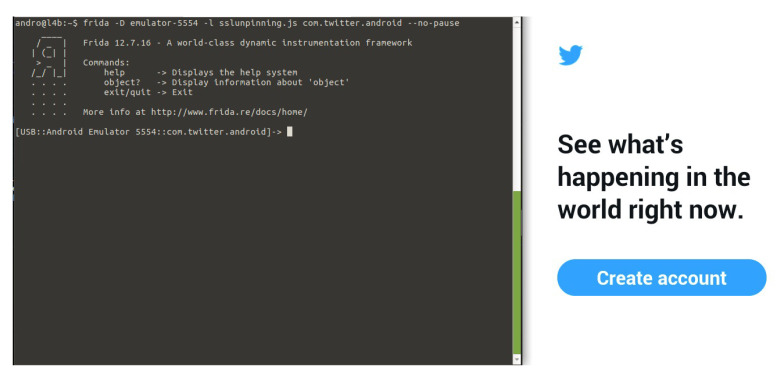
Frida intercepting the twitter app execution.

To perform this method, it is necessary to use a rooted mobile phone and a remote host. The process is described below:

1. The mobile phone must execute a server that acts as a dynamic code interpreter. The server acts as a daemon and it enables interacting dynamically with the physical memory of the device to manipulate and change the behaviour of the executing apps. Thus, the aim of the server is to manipulate or change the expected behaviour of the SSL pinning process. In our case, we used Frida server with the following command:
  (1) adbshell./serverscript.sh
2. It should be confirmed that the daemon server is running and has enough permissions to read and manipulate the memory of the device. With this aim, Frida framework can be used by means of the following command:
  (2) frida-ps-U
  Figure 6 shows how a Frida server is running in a mobile emulator.
3. Once the daemon server is running on the device, this fact enables the access to the physical memory of the device. This way, we are able to make the appropriate changes in memory to circumvent SSL Pinning measures. It is necessary to indicate which part of the app code must be dynamically modified in memory. To do that, a script must run on the server. In the following, we describe the steps of a script which modifies dynamically in the memory the code. Thus, the target app must be executed on the mobile together with the script to modify its code dynamically. There are several scripts (https://codeshare.frida.re/@pcipolloni/universal-android-ssl-pinning-bypass-with-frida/) that may be used. In our case, the script loads our certificate, creates a KeyStore containing our certificate and generates a TrustManager that trusts the certificate inside our KeyStore. When the app initialises its SSLContext, the parameter TrustManager in the *SSLContext.init()* method is swapped with the one we had created before.
4. It must be checked that there are not any restrictions that prevent the SSL handshake of the connection. If so, the connection is established. Finally, it must be confirmed that the target app works properly and SSL pinning was disabled. Figure 7 shows an example of how the execution of the Twitter app is intercepted by Frida.

If the app is implementing Root Detection, it is not possible to use Method 6, and Method 7 is used instead.

#### 4.3.2. Method 7: Dynamic Debugging of the App in Non-Rooted Devices

To perform this method, it is not necessary to use a rooted mobile. Steps 1–3 are performed considering that there is no point in rooting the device, as root detection countermeasure is implemented. Hence, it is necessary to skip this countermeasure to disable SSL Pinning. With this aim, the server daemon used in Method 6 must be embedded inside the application. The process is described below:

1. The target app code must be decompiled. For instance, *apktool* may be used.
2. A daemon must be embedded inside the target app. In our case, the daemon is Frida gadget. The function of the daemon is similar to Step 1 in Method 6.
  a. To put in the Frida gadget, an internet permission must be added to the Android manifest. Thereafter, Frida is able to open a socket.
  b. The file containing Frida libraries must be included to the app’s library folder.
  c. The onCreate method in the main activity of the target app must be modified to force the daemon to be run when the target app starts.
3. The modified app must be re-installed, launched, and its functioning must be checked.
4. From here, Steps 2–4 of Method 6 are executed.

**Figure 8:**
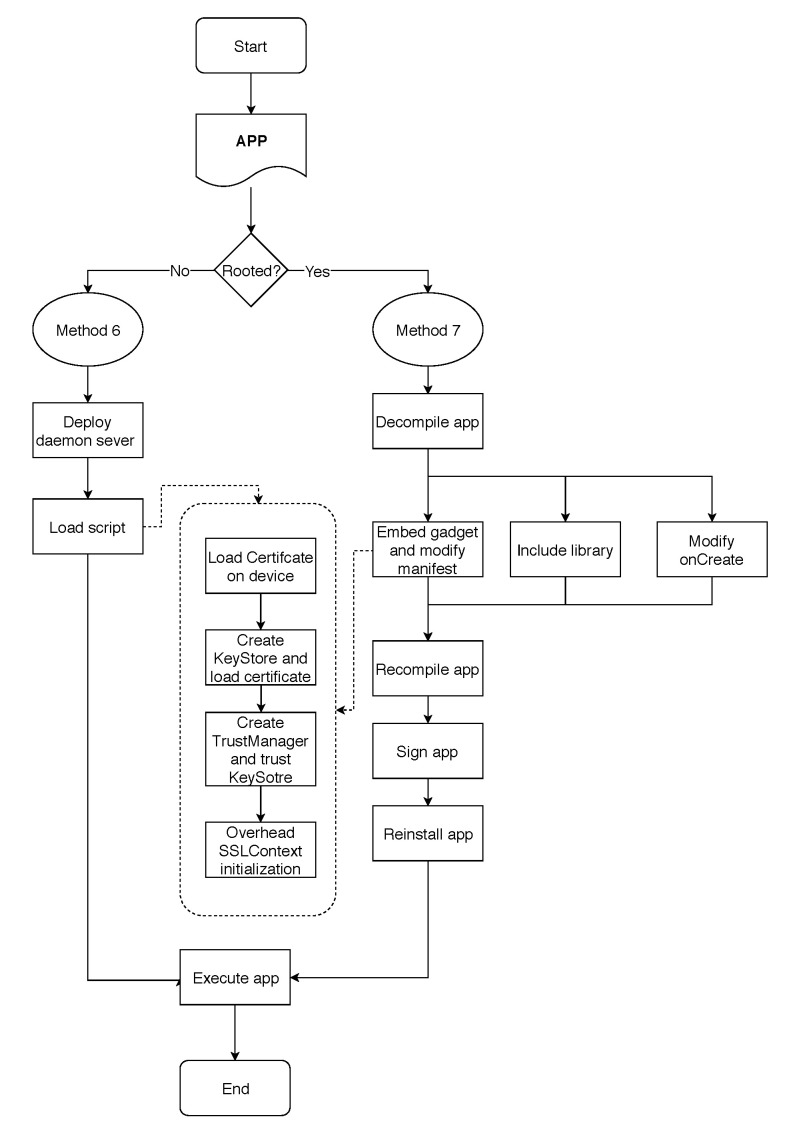
Functioning of SSL Pinning bypassing Methods 6 and 7.

In summary, Figure 8 shows a diagram describing the functioning of Methods 6 and 7.

### 4.4. Experiment Design

To evaluate our proposal, an experiment was designed. Our experiment is an adaptation of D’Orazio and Choo’s study [7] presented in Table 3. However, their study neither includes the analysis of security controls nor uses the Android version of the apps. The experiment consists of two phases: (1) checking whether the app includes SSL pinning and contains any security controls (see Section 3); and (2) checking the seven methods presented in the two previous sections. The first phase of our experiment is another novel contribution, while the second phase introduces two new bypassing methods.

To carry out the the first phase of the experiment and to check Methods 1–5, apktool [36], d2j-apk-sign [37], logcat [38] and SSL Unpinning [39] were used. These tools enable changing the certificates and debug the apps.

To perform the new proposal methods, we used Frida toolkit [40]. Frida is a framework designed for developers, security researchers and people who work in reverse engineer apps. It enables the hooking of processes to modify the code in a dynamic way. This allows injecting scripts written in JavaScript to perform certain operations in the app. We used Frida as a server to interact with the apps. To explore app executions, Objection was used. Objection [41] is a toolkit that enables the exploration of mobile devices at runtime. Objection toolkit was used to explore the execution of the apps.

The set of apps we used are the Android version of the apps used in D’Orazio and Choo’s study. These apps are ten widely used applications in different fields. Nevertheless, two of the apps (i.e., ANZ GoMoney and CopyApp) were substituted by two similar or more updated ones, Outlook and Instagram. We decided to change those two apps as they are not available in an Android version or their use in our region has been restricted.

## 5. Analysis of Results

In the next subsection, we present and analyse the results obtained for the experiments previously described. First, we checked which security controls were implemented by the apps. Next, we tried to bypass SSL pinning using the seven methods introduced above. Finally, we built a framework of applicability, relating the implemented security controls and the methods that are applicable.

### 5.1. SSL Pinning and Security Controls

In Table 4, we show the results of checking if the apps contain SSL pinning mechanisms. Moreover, we also checked whether the apps contain the security controls described in Section 3.

By analysing the results, the most outstanding facts are: (1) all apps implement SSL pinning mechanism; (2) six of the ten apps implement root detection and tamper detection mechanisms (in fact, they are the same ones); and (3) half the apps implement debug detection or obfuscation security controls. From the security point of view, we can consider Bank of America, NAB Bank app and WhatsApp the most secure apps, since they implement all the security controls defined in Section 3. Facebook and Instagram are the second best, despite not implementing the code obfuscation security control. PayPal and MEGA only implement SSL pinning. The rest of the apps implement two or three security controls.

### 5.2. SSL Pinning Bypassing Methods

Table 5 provides the results of checking the SSL pinning bypassing methods. We use ✓ symbol to indicate the correct bypassing and dash symbol otherwise. For the five old methods, we checked only the methods that appear as bypassed in Table 3, as we assume that the other methods are not applicable. On the other hand, for the new apps (i.e., Outlook and Instagram), all methods were checked.

By comparing the results for the old methods with the results in Table 5, there are very interesting facts to remark. The results for the iOS version of apps for Methods 1–3 remain equal. All apps were bypassed in the iOS versions using Methods 4 and 5. Nevertheless, only NAB Bank app, Bank of America and WhatsApp Android versions are not bypassed with Method 5. Those apps, together with Facebook and Instagram are not bypassed by Method 4.

Looking at the results for the new proposed methods, we can see how most of the apps that were vulnerable to the old methods are also bypassed with the new ones. There are other apps where the new methods fail to bypass SSL pinning, such as in the case of the Bank of America, NAB Bank app, Facebook, Instagram and WhatsApp.

In summary, we checked SSL bypassing with the five old methods, and we introduced two new bypassing methods.

### 5.3. Framework of Applicability

Based on the results shown in Table 5 and after analysing the experiments, we conclude that the framework of applicability is a key contribution of our research. This framework intends to help developers and auditors correctly develop and check secure communications, being a useful complement for the OWASP guidelines. Our framework helps simplifying the process of development and auditing Android mobile apps.

The framework of applicability is presented in Table 6. Each table cell indicates which SSL pinning methods are applicable depending on the implemented security control. Likewise, this table represents the impossibility of performing the SSL Pinning bypassing methods indicated by the ⇌ symbol when the security controls in the first column are implemented. In the following, we explain in detail each entry of the table from the point of view of security controls:

- *Root detection* disables Methods 3 and 6 when it is implemented. This fact occurs as the device needs to be rooted in some of the steps taken in the application of the methods.
- *Anti-tampering* security control disables the application of Methods 2, 5 and 7. This fact occurs as it is necessary to modify statically the app code to bypass the SSL pinning checks. This step requires to recompile the app and sign it. However, as we do not have access to the original certificate of the app, it is detected by the anti-tampering control.
- *Anti-debugging* security control stops Mmethods 3, 4, 6 and 7. As previously mentioned, a device in debug mode is detected, so the execution of the pinning is stopped since this measure stops step-by-step execution of the app code.
- *Code obfuscation* avoids Method 5 since obfuscation prevents the search of the functions that check the SSL Pinning.

On the other hand, we can read the table from point of view of the methods:

- *Method 2* can be tackled using the anti-tampering security control.
- *Method 3* can be stopped using root detection or anti-debugging controls.
- *Method 4* can be prevented with the anti-debugging control.
- *Method 5* can be stopped with the utilisation of anti-tampering or code obfuscation.
- *Method 6* can be held with the root detection and anti debugging security controls.
- *Method 7* can be denied with the anti-tampering and anti debugging controls.

This fact shows the need of implementing all the proposed security controls to stop all the SSL Pinning bypassing methods. It is remarkable that Method 1 is unrelated in the framework of applicability. Method 1 proves to be obsolete due to fact that it is easy to implement and most of the app developers may avoid it by changing the way of checking the certificate. Thus, Method 1 is, in practice, inoperative. In 2019, after the evolution in SSL pinning checking, changing CM in a certificate cannot be considered in SSL pinning bypassing, hence it is not applicable in any case. Moreover, we should point out that, even when a security control is not bypassed, we cannot guarantee that the control is completely secure. There is a certain possibility that a security control may be bypassed if the attacker has enough time. The auditor or the developer should evaluate the potential risk of SSL bypassing.

The framework of applicability provides a guideline with two main objectives: (1) learning the SSL pinning bypassing methods to check when some security controls are implemented; and (2) learning the security controls to implement against SSL pinning bypassing methods. Thus, the table can be read in two directions depending on the perspective to be used. For instance, if a pentester wants to check some security controls, the framework provides the SSL pinning bypassing methods to be used. On the other hand, if a developer wants an app to get protected against different SSL pinning bypassing methods, a set of security controls should be implemented.

In summary, the framework includes two dimensions: security controls and methods to circumvent SSL pinning. The first dimension describes the proposed methods to enforce the security of the SSL pinning mechanism from the point of view of the application developers. The second dimension deals with the methods to bypass SSL pinning depending on the scenario of application and the implemented controls. The second dimension is useful from the point of view of the auditors. The results of studying a set of apps regarding the security controls and methods returns the framework of applicability shown above. The experimentation supported the results given in Table 6. For instance, the cases of the apps Bank of America and WhatsApp demonstrate that the correct implementation of all security controls prevents the bypassing in any of the methods we applied. Otherwise, the incorrect application of security controls open a door to apply a set of methods to circumvent SSL pinning.

## 6. Conclusions

Nowadays, the use of mobile technologies has grown exponentially. The market for mobile apps is producing a new level of security threats since most of the apps require communications with external services. In fact, insecure communication ranks #3 in OWASP Top 10, so it is also quite an important topic to be considered. Currently, the use of apps is at least as common as consulting Internet. It is crucial to secure these apps to guarantee the information confidentiality and integrity. In this respect, we provide to app developers and pentesters with a guideline to enforce security in the communications of Android apps.

In this paper, we show how SSL/TLS implementations are vulnerable even when using SSL pinning techniques. This way, some measures have to be taken to protect an app from SSL pinning bypassing. In the implementation phase of the app, root detection, debug detection and anti-tampering measures must be implemented. Finally, the code must also be obfuscated.

We also study the methods that can bypass these countermeasures. First, we study the current bypassing SSL pinning techniques. Bypassing techniques are quite well-known in the professional world, as a business line for security auditors and consultants, but it is unusual to see them in academia. Thus, we try to fill that research gap. The main contributions and value of this paper are the introduction of five known methods, the addition of two new methods for circumventing SSL pinning, the presentation of some solutions that developers can use to avoid this and the development of a framework of applicability. This framework can be useful as a guideline for both developers and pentesters.

Five methods are introduced: change CM in certificate, insert CA in the app, use SSL Unpinning, debugging app and modify app executable. Then, we propose two new methods to bypass SSL/TLS validations in Android devices. The first method deals with rooted devices and the second one deals with non-rooted devices. Finally, we evaluated our proposal, with the design of an experiment with a set of apps. The experiment consisted of two phases: (1) checking whether the app includes SSL pinning and contains any security controls; and (2) checking the seven methods presented in the paper. The set of apps we used consists of ten widely used applications in different fields.

The results demonstrate whether the Android version of the app can implement the same security controls as the iOS version. Moreover, the new bypassing methods can be useful as an addition to the older ones.

Finally, we propose a framework of applicability as a key contribution of our research. This framework intends to help developers and auditors for the correct development and checking of secure communications, being a useful complement for the OWASP guidelines. Our framework helps simplify the process of developing and auditing Android mobile apps. The framework of applicability provides a guideline with two main objectives: (1) learning the SSL pinning bypassing methods to check when some security controls are implemented; and (2) learning the security controls to implement against SSL pinning bypassing methods. This framework is useful both from the point of view of the developer and the security auditor. In addition, the framework of applicability shows the need of implementing all the proposed security controls to stop all the SSL Pinning bypassing methods. An evaluation of the framework of applicability will be part of our further research.

It has been demonstrated how SSL pinning can be circumvented, attacking the integrity of communications. We provide some mechanisms to prevent these attacks.

## Figures and Tables

**Table 1:** OWASP Top 10.

Category	Name
M1	Improper Platform Usage
M2	Insecure Data Storage
M3	Insecure Communication
M4	Insecure Authentication
M5	Insufficient Cryptography
M6	Insecure Authorisation
M7	Client Code Quality
M8	Code Tampering
M9	Reverse Engineering
M10	Extraneous Functionality

**Table 2:** Network communication security verification requirements.

Control	Description
5.1	Data are encrypted on the network using TLS. The secure channel is used consistently throughout the app
5.2	The TLS settings are in line with current best practices, or as close as possible if the mobile operating system does not support the recommended standards
5.3	The app verifies the X.509 certificate of the remote endpoint when the secure channel is established. Only certificates signed by a trusted CA are accepted
5.4	The app uses its own certificate store, or pins the endpoint certificate or public key, and subsequently does not establish the connection with endpoints that offer a different certificate or key, even if signed by a trusted CA
5.5	The app does not rely on a single insecure communication channel (email or SMS) for critical operations, such as enrollments and account recovery

**Table 3:** Summary of SSL/TLS apps bypassed by D’Orazio and Choo [7].

App	Principal Method				
	Method 1	Method 2	Method 3	Method 4	Method 5
Twitter	-	-	-	✓	✓
NAB Bank app	-	-	-	✓	✓
Dropbox	-	-	✓	✓	✓
WhatsApp	-	-	✓	✓	✓
Bank of America	-	-	-	✓	✓
Facebook	✓	✓	-	✓	✓
ANZ GoMoney	-	-	-	✓	✓
PayPal	✓	-	-	✓	✓
MEGA	-	-	-	✓	✓
CopyApp	✓	✓	-	✓	✓

**Table 4:** Results of checking SSL pinning and controls.

Name App	SSL Pinning	Root Detection	Tamper Detection	Debug Detection	Obfuscated
Twitter	✓	-	-	-	✓
NAB Bank app	✓	✓	✓	✓	✓
Dropbox	✓	-	-	-	✓
WhatsApp	✓	✓	✓	✓	✓
Bank of America	✓	✓	✓	✓	✓
Facebook	✓	✓	✓	✓	-
Outlook	✓	✓	✓	-	-
PayPal	✓	-	-	-	-
MEGA	✓	-	-	-	-
Instagram	✓	✓	✓	✓	-

**Table 5:** Results of bypassing SSL pinning methods.

App	Principal Method					New Methods	
	Method 1	Method 2	Method 3	Method 4	Method 5	Method 6	Method 7
Twitter	-	-	-	✓	✓	-	✓
NAB Bank app	-	-	-	-	-	-	-
Dropbox	-	-	✓	✓	✓	✓	✓
WhatsApp	-	-	-	-	-	-	-
Bank of America	-	-	-	-	-	-	-
Facebook	-	-	-	-	✓	-	-
Outlook	-	-	-	✓	✓	-	✓
PayPal	-	-	-	✓	✓	✓	✓
MEGA	-	-	-	✓	✓	✓	✓
Instagram	-	-	-	-	✓	-	-

**Table 6:** Framework of applicability methods vs. security controls.

	Method 1	Method 2	Method 3	Method 4	Method 5	Method 6	Method 7
Root Detection			⇌			⇌	
Anti tampering		⇌			⇌		⇌
Anti debugging			⇌	⇌		⇌	⇌
Obfuscation					⇌		
